# DNA Damage, Somatic Aneuploidy, and Malignant Sarcoma Susceptibility in Muscular Dystrophies

**DOI:** 10.1371/journal.pgen.1002042

**Published:** 2011-04-14

**Authors:** Wolfgang M. Schmidt, Mohammed H. Uddin, Sandra Dysek, Karin Moser-Thier, Christine Pirker, Harald Höger, Inge M. Ambros, Peter F. Ambros, Walter Berger, Reginald E. Bittner

**Affiliations:** 1 Neuromuscular Research Department, Center of Anatomy and Cell Biology, Medical University of Vienna, Vienna, Austria; 2 Institute of Cancer Research, Department of Medicine I, Medical University of Vienna, Vienna, Austria; 3 Division for Laboratory Animal Science and Genetics, Medical University of Vienna, Himberg, Austria; 4 Children's Cancer Research Institute (CCRI), St. Anna Kinderkrebsforschung Association, Vienna, Austria; University of Minnesota, United States of America

## Abstract

Albeit genetically highly heterogeneous, muscular dystrophies (MDs) share a convergent pathology leading to muscle wasting accompanied by proliferation of fibrous and fatty tissue, suggesting a common MD–pathomechanism. Here we show that mutations in muscular dystrophy genes (*Dmd*, *Dysf*, *Capn3*, *Large*) lead to the spontaneous formation of skeletal muscle-derived malignant tumors in mice, presenting as mixed rhabdomyo-, fibro-, and liposarcomas. Primary MD–gene defects and strain background strongly influence sarcoma incidence, latency, localization, and gender prevalence. Combined loss of dystrophin and dysferlin, as well as dystrophin and calpain-3, leads to accelerated tumor formation. Irrespective of the primary gene defects, all MD sarcomas share non-random genomic alterations including frequent losses of tumor suppressors (*Cdkn2a*, *Nf1*), amplification of oncogenes (*Met*, *Jun*), recurrent duplications of whole chromosomes 8 and 15, and DNA damage. Remarkably, these sarcoma-specific genetic lesions are already regularly present in skeletal muscles in aged MD mice even prior to sarcoma development. Accordingly, we show also that skeletal muscle from human muscular dystrophy patients is affected by gross genomic instability, represented by DNA double-strand breaks and age-related accumulation of aneusomies. These novel aspects of molecular pathologies common to muscular dystrophies and tumor biology will potentially influence the strategies to combat these diseases.

## Author Summary

All kinds of muscular dystrophies (MDs) are characterized by progressive muscle wasting due to life-long proliferation of precursor cells of myo- (muscle), fibro- (connective tissue), and lipogenic (fat) origin. Despite discovery of many MD genes over the past 25 years, MDs still represent debilitating, incurable diseases, which frequently lead to premature death. Thus, it is imperative to gain novel insights into the underlying MD pathomechanisms. Here, we show that different mouse models for the most common human MDs frequently develop skeletal musculature-associated tumors, presenting as complex sarcomas, consisting of myo-, lipo-, and fibrogenic compartments. Collectively, these tumors are characterized by profound genomic instability such as DNA damage, recurring mutations in cancer genes, and aberrant chromosome copy numbers. We also demonstrate the presence of these cancer-related aberrations in dystrophic muscles from MD mice prior to formation of visible sarcomas. Moreover, we discovered corresponding genomic lesions also in skeletal muscles from human MD patients, as well as stem cells cultured thereof, and show that genomic instability precedes muscle degeneration in MDs. We thus propose that cancer-like genomic instability represents a novel, unifying pathomechanism underlying the entire group of genetically distinct MDs, which will hopefully open new therapeutic avenues.

## Introduction

*“… fibres were found to be completely destroyed, the sarcous element being diffused, and in many places converted into oil globules and granular matter…”*

(Edward Meryon, 1852)

Muscular dystrophies (MDs) comprise a group of inherited disorders, characterized by progressive muscle wasting and weakness, frequently causing premature death due to lack of effective therapies. More than 150 years ago, Edward Meryon was the first to characterize the detrimental “fatty degeneration of the voluntary muscles” in Duchenne MD (Meryon E. *Lancet* 2:588, 1851). Today it is well accepted that the progressive loss of functional muscle tissue and its replacement by adipose and fibrous tissue represent a pathology common to all MDs despite their heterogeneous genetic etiology. Most MDs are caused by gene mutations that lead to absence or dysfunction of structurally and/or functionally important molecules of the muscle fiber [1], [2]. The sarcoplasmic spectrin-related protein dystrophin is thought to structurally stabilize the muscle fiber sarcolemma by linking the actin-based cytoskeleton to the extracellular matrix via interaction with the dystroglycan (DG)-complex. Lack or vast reduction of dystrophin causes severe Duchenne muscular dystrophy (DMD) [3] in humans and myopathy in corresponding mouse models, such as the *mdx* [4] or *mdx*-*3Cv* [5] mouse. Mutations in several glycosyltransferase-encoding genes, such as the fukutin related protein (*FKRP*) or *LARGE* lead to defective glycosylation of the α-subunit of DG. This molecular defect underlies the second most common group of MDs, the so called “secondary dystroglycanopathies”. Numerous other MD-related molecules are not known to directly interact with the DG complex, such as dysferlin or calpain-3. Defective expression of dysferlin, a ubiquitously expressed 230-kDa transmembrane protein that has been shown to be involved in resealing muscle fiber membranes, causes limb-girdle muscular dystrophy type 2B (LGMD2B) or Miyoshi-myopathy in humans [6], [7]. An inbred mutation in the murine dysferlin (*Dysf*) gene makes the *SJL*-mouse a naturally occurring animal model for the human dysferlinopathies [8]. Mutations of the *CAPN3* gene encoding the muscle-specific calcium-activated neutral protease calpain-3, a proteolytic switch in muscle remodeling [9], cause LGMD2A, a MD with a wide clinical spectrum [10]. Again, the corresponding animal model, the *Capn3*-deficient mouse is only affected by a mild progressive muscular dystrophy [11]. Given the diverse and obviously unrelated functions of these proteins, whose absence or dysfunction causes MDs, a common pathomechanism driving the complex events of parallel muscle regeneration and degeneration and progressive proliferation of fibrous and fatty tissue seen in all MDs is likely but still remains elusive. In the light of the fact that nearly 25 years ago the *DMD* gene was identified as the molecular basis for Duchenne MD, the lack of causative therapies has dampened earlier therapeutic promises based on the discovery of molecular defects underlying several MDs and underscores the imperative need for a comprehensive understanding of pathology involved in these rare but lethal diseases.

When starting to study age-related phenotypes of murine MDs, we have observed the frequent and spontaneous occurrence of skeletal muscle-derived tumors in our colony of C57BL/10-*mdx* mice, suggesting a tumor-suppressive role of dystrophin in mice. Therefore we extended our studies to other dystrophin mutations, mouse strains, and even to other MD-mouse models for the most frequent MDs in humans, like dysferlin, calpain-3 and Large, respectively. We show that all of these MD-mouse lines are prone to develop mixed soft-tissue sarcomas containing tumor elements displaying histological and molecular characteristics of rhabdomyo-, fibro-, and liposarcoma. These MD-associated tumors share complex, non-random genomic alterations affecting well-known tumor suppressor as well as oncogenes and these cancer signatures are already detectable in dystrophic muscle tissue, independent of the underlying mutation. Consequently, we show that genomic instability and DNA damage are present also in muscle of human MD patients. Collectively, these data strongly support an unprecedented general link between muscular dystrophy and cancer, driven by the accumulation of DNA damage, chromosome copy number aberrations, and finally the origin of cell clones harboring cancer-like mutations in dystrophic muscle tissue. We propose that - similar to pre-neoplastic lesions - the dystrophic muscle is characterized by genomic instability, which contributes to a common hyperproliferative pathomechanism promoting the degenerative process in human MDs and favoring age-related tumorigenesis in the respective mouse models.

## Results

### Spontaneous occurrence of skeletal muscle-derived tumors in various dystrophin-deficient mouse lines

During the last two decades we have observed the spontaneous occurrence of soft tissue tumors arising from various skeletal limb and trunk muscles in our dystrophin-deficient C57BL/10 *mdx*-mouse [4] cohort. These tumors arose in aged *mdx* mice (mean age-of-onset: ∼540 d) with an incidence of almost 40%, whereas we never observed the occurrence of such tumors in our C57BL/10 wild-type mice. In our colony of another dystrophin-deficient mouse line, *mdx-3Cv*, which lacks both the muscle 427 kDa and non-muscle 71 kDa dystrophin isoforms due to a mutation at the intron-exon 66 junction [5], we observed the spontaneous occurrence of skeletal muscle-derived tumors indistinguishable from those observed in C57BL/10-*mdx* mice. However, *mdx-3Cv* developed skeletal muscle-tumors at a significant older age (∼660 d) and a decreased incidence of only 5% as opposed to our *mdx* colony. Because we could not figure out if these differences were due to the different genetic backgrounds (*mdx*: C57BL/10, *mdx-3Cv*: C57BL/6 x B6C3Fe) or due to the different dystrophin-mutations, we generated two novel *mdx* inbred strains, i.e. BALB/c-*mdx*, and C3H-*mdx*, respectively, and further studied *mdx*-mice on mixed C57BL/6 x BALB/c and C57BL/10 x B6C3Fe backgrounds. Indeed we observed the spontaneous occurrence of skeletal muscle-tumors also in these *mdx*-mice, underlining a strain-independent tumor-suppressor role of dystrophin. Mean ages-of-onset, incidences and gender distributions of tumor-formation were strongly strain-dependent, whereby the C57BL/10 background was most tumor-susceptible (Table 1). The spontaneous occurrence of skeletal muscle-associated tumors in different dystrophin-deficient mouse lines independent of the underlying dystrophin gene mutation supported a candidate tumor suppressor role of dystrophin.

**Table 1:** Incidence and age-of-onset of malignant sarcomas in MD mouse models.

Genotype	Strain background	Cohort	Sarcomas	Incidence^a^	Mean age	[CI (95%)]	Female/male ratio	Tumor site predilection
**Dystrophin**								
*Dmd ^−/−^ (mdx)*	C57BL/10	n = 122	48	39%	539 d	[516–562]	1: 1.1	proximal hind limb, trunk, head & neck
	C57BL/10× B6C3Fe^b^	n = 46	8	17%	580 d	[460–700]	1.7: 1	proximal hind limb, trunk, head & neck
	C57BL/6× BALB/c^c^	n = 99	10	10%	651 d	[567–735]	1: 1.4	proximal hind limb, fore limb, head & neck
	BALB/c	n = 82	7	9%	515 d	[413–607]	1: 1.3	proximal hind limb, head & neck
	C3H	n = 60	9	15%	523 d	[411–635]	2.5: 1	proximal hind limb, trunk, head & neck
*Dmd ^−/−^(mdx-3Cv)*	C57BL/6× B6C3Fe^d^	n = 55	3	5%	663 d	n.a.	n.a.	n.a.
**Dysferlin**								
*Dysf ^−/−^*	C57BL/10	n = 151	35	23%	640 d	[601–679]	1: 2.9	proximal hind limb and trunk
	C57BL/10× B6C3Fe^b^	n = 78	17	22%	755 d	[686–804]	1: 2.9	trunk
**Large**								
*Large ^−/−^ (myd*)	B6C3Fe^c^	n = 71	1	1%	278 d	n.a.	n.a.	n.a.
**Calpain**								
*Capn3 ^−/−^*	C57BL/6× [129/Sv: C57BL/6]^d^	n = 19	1	5%	716 d	n.a.	n.a.	n.a.
**Dystrophin-Dysferlin**								
*Dmd ^−/−^ Dysf ^−/−^*	C57BL/10	n = 89	42	47%	389 d	[369–409]	1: 2.4	proximal hind limb, fore limb, trunk, head & neck
**Dystrophin-Calpain**								
*Dmd ^−/−^ Capn3 ^−/−^*	C57BL/10× [129/Sv: C57BL/6]^ b^	n = 55	24	44%	391 d	[360–422]	1: 1.6	proximal hind limb, trunk

**a** Incidences of clinically overt sarcomas. Different C57BL/6 proportions in mixed backgrounds are indicated: **b** 25%, **c** 50%, and **d** 75% C57BL/6.

n.a.: not applicable.

### Mice mutated in dysferlin, calpain-3, and Large are also prone to develop skeletal muscle-derived sarcomas

**Figure 1:**
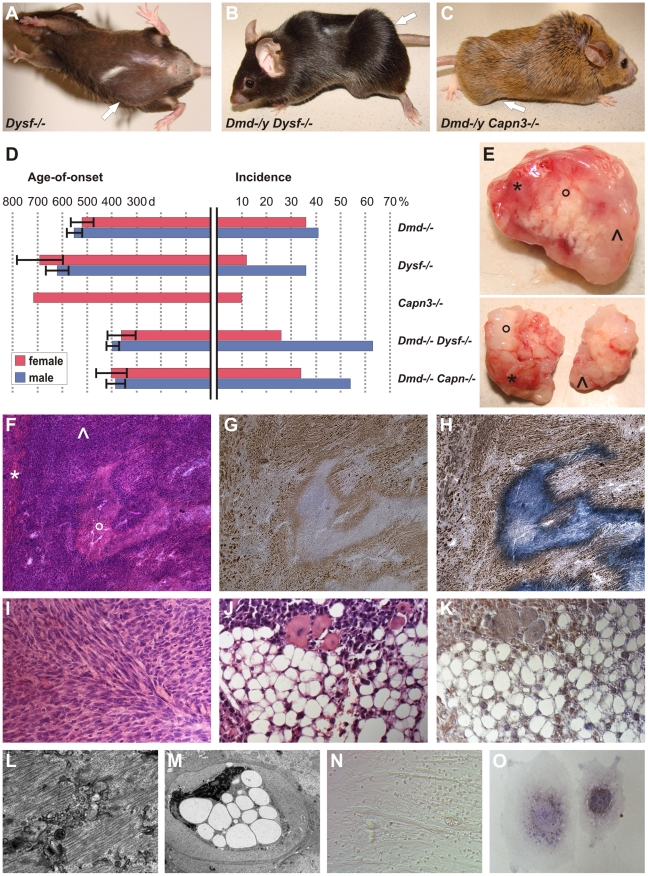
MD mice are prone to develop skeletal muscle-related malignant mixed mesenchymal tumors. (A) Dysferlin-deficient mouse (male, 878 d) with sarcoma located in the caudal abdominal wall (arrow; weight of excised tumor: 4.2 g). (B) Dystrophin-dysferlin double-mutant (male, 250 d) with very large sarcoma (9.1 g), which arose in proximal and distal skeletal muscles of the left hindlimb (arrow). (C) Dystrophin-calpain 3 double-mutant (male, 474 d) with sarcoma (2.3 g) located in ischiocrural muscles of the right hindlimb (arrow). (D) Graphical representation of sarcoma incidence and age-of-onset from life-span studies of mice lacking dystrophin (n = 48/122; C57BL/10), dysferlin (n = 35/151; C57BL/10), or calpain 3 (n = 1/19; 129/Sv x C57BL/6) and of dystrophin-dysferlin (n = 42/89) and dystrophin-calpain 3 (n = 24/55) double-mutant mice. Error bars indicate confidence interval of the mean (95%). (E) Representative examples of excised tumors (from dystrophin-dysferlin double-mutant mice) with myo- (*), lipo- (°), and fibrosarcomatous (^) compartments macroscopically recognizable as different parts of the tumor mass. (F–H) Histological examination of the tumor shown in E (upper panel) revealed a sarcoma with mixed mesenchymal differentiation characterized by rhabdomyo- (*), lipo-(°), and fibrosarcoma (^) components. Serial sections used for H&E staining (F), myogenin staining (G), and double staining with myogenin and Sudan Black (H). (I) Morphological analysis of a sarcoma that arose in a 486 d-old C57BL/10 *mdx* mouse revealing a prominent fibrosarcoma areal characterized by the typical collagen fibres bundles arranged in “herringbone pattern” (H&E). (J,K) Well-differentiated liposarcoma area within a mixed sarcoma from a *Dmd ^−/−^ Capn3 ^−/−^* double-mutant mouse (J, H&E), with intense Cdk4-positivity (K). (L) Electron micrograph showing a rhabdomyosarcoma cell displaying myofibril structures. (M) Electron micrograph from the same tumor showing a liposarcoma cell densely packed with large lipid vesicles and exhibiting a highly aberrantly shaped nucleus. (N) Photomicrograph showing cultured tumor cells propagated from a typical mixed sarcoma (from a dysferlin-deficient mouse), indicating the presence of different types of tumor cells (round-shaped, spindle cell-like, and myotube-like elongated rhabdomyoblasts). (O) Photograph of two tumor cells grown *in vitro* from the same explanted tumor. Double-staining for myogenin and Sudan Black depicted a myogenin-positive myogenic tumor cell next to a myogenin-negative but lipogenic tumor cell containing Sudan Black-positive droplets.

In order to learn whether other MD-genes, which are not directly related to dystrophin, might also suppress tumor formation, we studied mice lacking dysferlin (*Dysf^SJL^* mutation [8]; *Dysf ^−/−^*), calpain-3 (*Capn3 ^−/−^*; knockout [11]), or Large (*Large^myd^* mutation [12]; *Large ^−/−^*). In a colony of *Dysf ^−/−^* mice inbred onto C57BL/10 (n = 151), we also observed high incidence (23%; male-to-female ratio ∼3:1) of age-related sarcomas (∼640 d), which mainly arose from proximal hind limb muscles. Also for *Dysf ^−/−^* mice a strain-dependent effect with respect to mean age of sarcoma-onset was detected, which was more than 100 days later (∼755 d) when the mutation was bred on a mixed C57BL/10 x B6C3Fe background, whereas the sarcoma incidence remained unchanged (22%). Notably, dysferlin-deficiency on the mixed C57BL/10 x B6C3Fe background resulted in a predominant abdominal wall location of sarcomas (Figure 1A, Table 1).

Based on the spontaneous occurrence of skeletal muscle-tumors in mice deficient for the so far molecularly unrelated genes dystrophin and dysferlin, we hypothesized that MD-genes in more general might act as tumor suppressors. To this end, we conducted a life-span study with mice lacking calpain-3, the animal model for LGMD2A in humans. Indeed, also *Capn3 ^−/−^* mice developed skeletal muscle-derived sarcomas at an incidence of 5%. Finally, we also observed the rare occurrence of sarcoma formation even in *myd* mice (representing a model for a severe congenital MD in humans), in spite of their considerably short lifespan (Table 1).

### Combined defects in MD genes accelerate sarcomagenesis in mice

In order to test if dystrophin, dysferlin and calpain-3 have tumor-suppressor effects *in vivo*, we generated double-mutant mouse lines, i.e. dystrophin-deficient (*mdx*) mice with additional lack of either dysferlin (*Dmd ^−/−^ Dysf ^−/−^*) or calpain-3 (*Dmd ^−/−^ Capn3 ^−/−^*). *Dmd ^−/−^ Dysf ^−/−^* mice (C57BL/10) clinically presented with significant weakness characterized by severe dystrophic signs in the skeletal muscle (R.B., *manuscript in preparation*), and had a severely reduced life-span of ∼13 months. Remarkably, malignant skeletal muscle-derived sarcomas (Figure 1B) constituted the main cause of premature death in this condition. While penetrance was sharply increased in male mice, 63% of which developed sarcomas, a dramatic decrease in tumor latency was observed in both genders, with the mean age-of-onset reduced to ∼390 d (compared to 540 d in *Dmd ^−/−^* and 640 d in *Dysf ^−/−^*).

The combined effect of *Dmd ^−/−^ Capn3 ^−/−^* in double-knockout mice, which also presented with a severe MD-phenotype leading to a shortened life-span of ∼13 months (R.B., *manuscript in preparation*), resulted in spontaneous sarcoma-formation in 44% of the animals with a mean-age of onset of ∼390 days (Figure 1C). Thus, additional loss of both dysferlin and calpain-3 in dystrophin-deficient *mdx* mice dramatically reduced sarcoma latency (Figure 1D).

### The skeletal muscle-derived tumors in MD mice present as mixed rhabdo-, fibro-, and liposarcomas

Because the macroscopic appearances of the skeletal muscle-derived tumors showed areas of different colorings and varying consistencies (Figure 1E), we speculated that this might be due to a mixed composition of diversely differentiated tumor-cell lineages. Indeed, careful histopathological examinations revealed that all tumors independent from the underlying MD mutation(s) resembled mixed sarcomas, comprising variably sized coexisting compartments of rhabdomyosarcoma (RMS), fibrosarcoma (FS) and liposarcoma (LS), respectively (Figure 1F–1K). Histopathology of RMS mainly presented as embryonic (ERMS) or spindle-cell tumors, which expressed myogenic factors to various degrees, such as myogenin (Figure 1G), Myf5, or desmin (*not shown*). Also at the ultrastructural level, these tumor compartments were composed of cells with myofibrils, which were partly arranged in sarcomeric manner (Figure 1L). Tumor-compartments identified as FS displayed bundles of collagen fibres and immature, proliferating fibroblasts, which were arranged in a typical herringbone pattern, hereby recapitulating the histopathological hallmark of human FS (Figure 1F, 1I). The third identifiable compartment was LS consisting of lipocytes, which showed both well-differentiated and de-differentiated morphologies. The unifying characteristics of all LS-cells were positivity for lipid staining by Sudan Black (Figure 1H) and, moreover, immunoreactivity for Cdk4 (a human LS biomarker) (Figure 1K). By electron microscopy, LS-cells characteristically contained numerous fat droplets (Figure 1M). In line with the histological findings, propagated tumor cell cultures also revealed co-existence of different cell types (Figure 1N), most prominently myogenin-positive cells and lipogenic but myogenin-negative cells (Figure 1O), providing further support that tumors arising in MD-mice are mixed-type sarcomas. Because these findings disclosed all MD-tumors as complex, mixed sarcomas, we next studied the expression of select human sarcoma-related genes [13]. Indeed we found increased expression levels for RMS-markers (*Myog*, *Myl4*, *Igf2, Prox1*), a FS-gene (*Vcan*), and LS-related genes (*Pparg*, *Myo1e*, *Hoxa5*, *Plau*), which further established the MD-tumors as mixed sarcomas consisting of RMS, FS and LS-compartments (Figure S1).

### Genomic hallmarks of murine MD sarcomas: amplification of *Met* and *Jun*, loss of *Cdkn2a* and *Nf1*, and whole chromosome 8 and 15 gains

**Figure 2:**
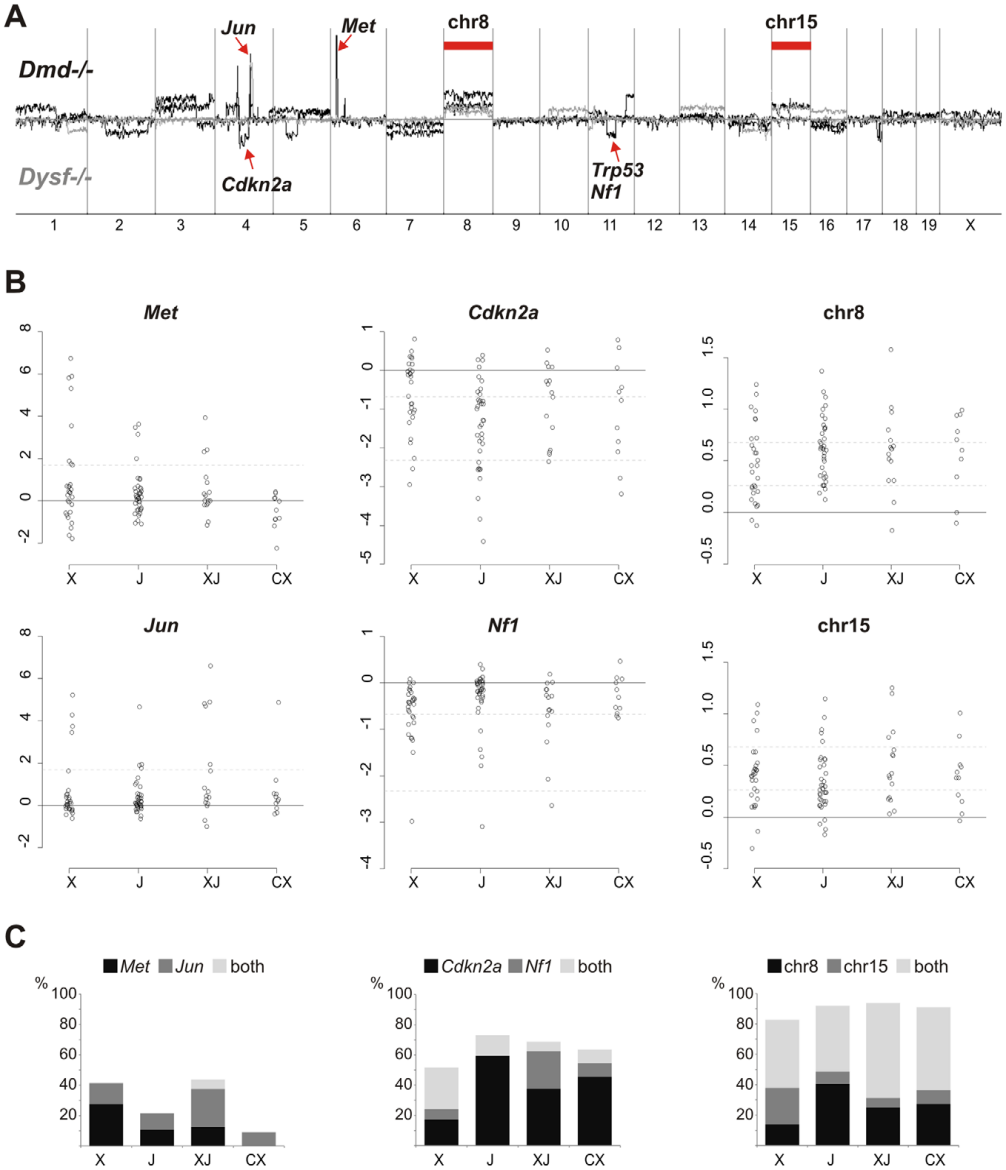
Common non-random genetic lesions in MD-mouse sarcomas. (A) Chromosomal aberration profiles from Agilent aCGH experiments performed on tumor DNA isolated from sarcomas of n = 4 *Dmd ^−/−^* (black) and n = 4 *Dysf ^−/−^* (grey) mice revealing gross genomic instability, specific losses at the *Cdkn2a*, *Nf1*, and *Trp53* tumor suppressor loci, amplification of *Met* and *Jun* oncogenes, and recurrent gains of whole chromosome 8 and 15. (B) Real-time PCR quantification of *Met* and *Jun* oncogene amplification, losses at the *Cdkn2a* and *Nf1* loci, and of chromosome 8 and 15 copy numbers in n = 98 sarcomas from *Dmd ^−/−^* (X), *Dysf ^−/−^* (J), *Dmd ^−/−^ Dysf ^−/−^* (XJ), and *Dmd ^−/−^ Capn3 ^−/−^* (CX) mice. Relative copy numbers (shown as log_2_) were calculated by the ΔΔC_t_-method. Dashed lines indicate log_2_-thresholds that were set under the assumption of 80% tumor cell content: gene amplification (4-fold; *Met* and *Jun*), deletions (first line for heterozygous losses, 0.5-fold, second line for homozygous losses; *Cdkn2a* and *Nf1*), and chromosome copy numbers (3 and 4). (C) Frequency plots for *Met* and *Jun* amplification, *Cdkn2a* and *Nf1* deletions, and chromosome 8/15 gains from the n = 98 sarcomas shown in (B).

In order to characterize the emerging link between MD and sarcoma susceptibility, we investigated genetic lesions in tumors originating in our MD-mouse strains. DNA extracted from solid tumors from *Dmd ^−/−^* or *Dysf ^−/−^* mice was subjected to an arrayCGH-based screen (n = 8), which revealed that the majority of *Dmd ^−/−^* tumors were characterized by multiple segmental chromosomal changes, chromosome number aberrations, and amplification of loci harboring the *Met* (encoding the Met proto oncogene hepatocyte growth factor receptor) or *Jun* oncogene, while tumors from *Dysf ^−/−^* mice typically displayed less genomic instability (Figure 2A). Frequent disruption of the tumor suppressor loci *Cdkn2a*, encoding p16^INK4a^ and p19^ARF^, *Nf1*, encoding neurofibromin 1, and *Trp53*, together with whole chromosome 8 and/or 15 gains represented key non-random alterations of sarcomas in both MD models.

Quantitative PCR (qPCR) experiments (Figure 2B and 2C) of DNA extracted from tumors of *Dmd ^−/−^*, *Dysf ^−/−^*, *Dmd ^−/−^ Dysf ^−/−^*, and *Dmd ^−/−^ Capn3 ^−/−^* mice (n = 98) revealed that these genetic lesions were common but occurred at different degrees, depending on the specific gene defect(s). Frequent amplification of *Met* or *Jun* oncogenes was observed in *Dmd ^−/−^* (41%) and *Dmd ^−/−^ Dysf* sarcomas (44%). In contrast, amplifications of *Mdm2* and/or *Cdk4* (which were additionally tested because of their frequent amplification in human sarcomas, most prominently liposarcomas [14]) were rare (<5%; *not shown*).

Lesions of the *Nf1* gene (exons 23 and/or 56) were more frequently found in *Dmd ^−/−^* (34%) and *Dmd ^−/−^ Dysf ^−/−^* sarcomas (31%) as opposed to *Dysf ^−/−^* (14%) or *Dmd ^−/−^ Capn3 ^−/−^* (18%). Conversely, exon 2 of the *Cdkn2a* tumor suppressor gene, which encodes parts of both p16^INK4a^ and p19^ARF^, was reduced in 73% of *Dysf ^−/−^* tumors whereas ∼50% of *Dmd ^−/−^ Dysf ^−/−^* and *Dmd ^−/−^ Capn3 ^−/−^* tumors carried this deletion. Notably, many of the qPCR-ratios obtained for *Cdkn2a* and *Nf1* were consistent with losses throughout the tumor. In 25% of DNA samples from sarcomas with qPCR values indicating *Cdkn2a* loss, exon 2 copy numbers were <0.2, which suggested the presence of a homozygous deletion in ∼80% of tumor cells, compatible with an early event in tumorigenesis.

Based on the arrayCGH-findings we screened a large cohort of tumors also for chromosome 8 and 15 copy number aberrations. We found gains of either or both chromosomes in the vast majority (80%) of sarcomas. While ∼40–60% of tumors from all MD models displayed gains of both chromosomes, chromosome 8 alone was preferably gained in *Dysf ^−/−^* and chromosome 15 in *Dmd ^−/−^* tumors indicating a probable MD-specific preference (Figure 2C).

### Sarcoma and dystrophic muscle display similar patterns of genomic instability

More than 50% of the measured chromosome 8/15 ratios were consistent with gains throughout the tumor, implying the presence of trisomies in more than 90% of tumor cells. This suggested that together with losses at *Cdkn2a* and *Nf1* loci the recurrent duplications of these chromosomes belong to early events in sarcoma development. Thus, we argued that such events might occur in skeletal muscles of MD-mice prior to formation of clinically identifiable tumors.

**Figure 3:**
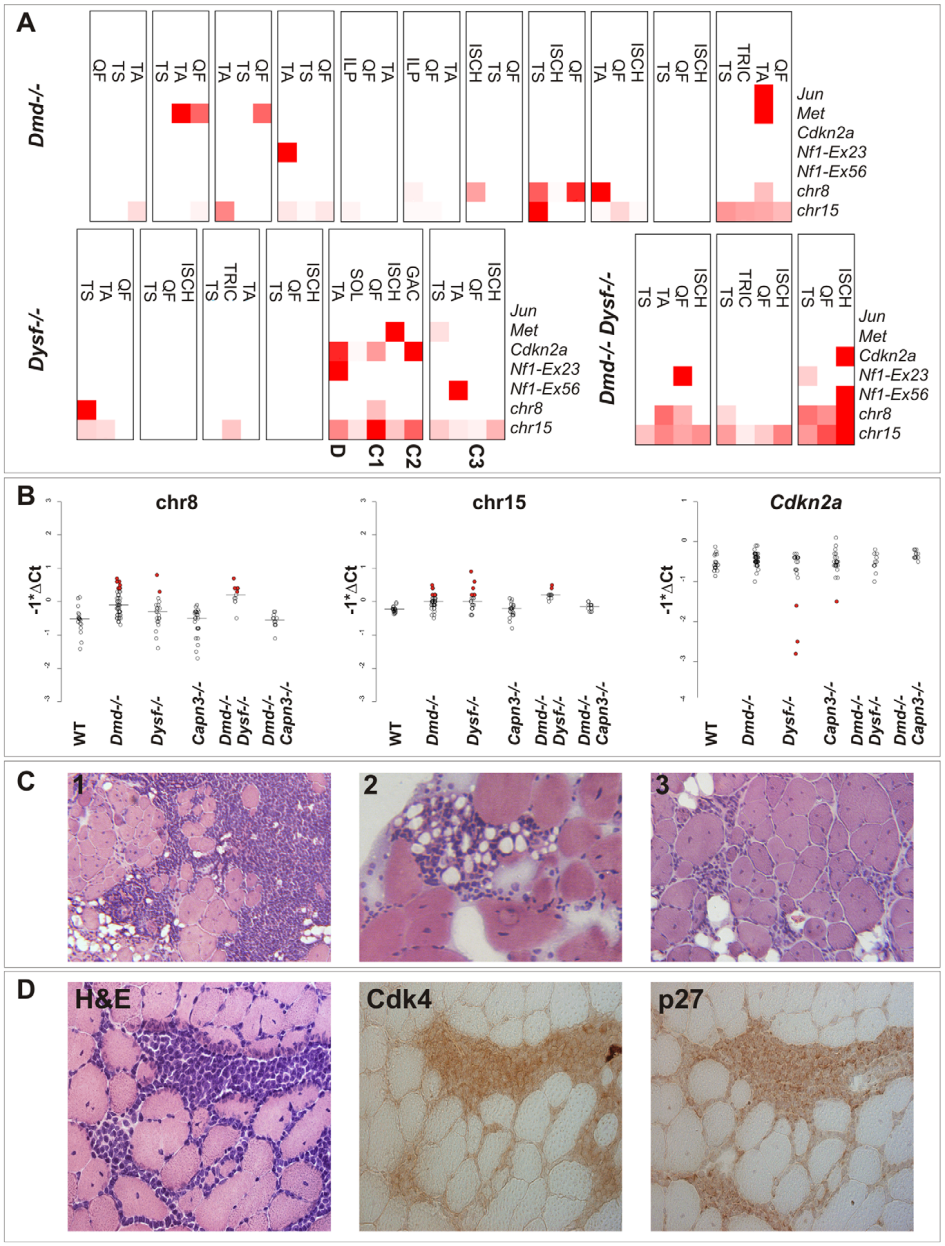
Genetic alterations frequently found in sarcomas are detectable in dystrophic skeletal muscle from clinically tumor-free mice. (A) Heatmap representation of real-time PCR results from quantification of *Jun*, *Met*, *Cdkn2a*, *Nf1* (exon 23 and 56, respectively), and chromosome 8 and 15 copy number in DNA samples extracted from tumor-prone skeletal muscles (QF: quadriceps femoris, TA: tibialis anterior, TRIC: triceps brachii, TS: triceps surae, ISCH: ischiocrural muscles, ILP: iliopsoas, GAC: gastrocnemius, SOL: soleus) from n = 20 different aged MD-mice (mean age of mice: *Dmd ^−/−^*: 590 d; *Dysf ^−/−^*: 640 d; *Dmd ^−/−^ Dysf ^−/−^*: 482 d) without clinically overt tumors (boxes indicate different individuals). qPCR measurements were highly suggestive of occasional copy number aberrations of the *Cdkn2a*, *Nf1*, *Met* and *Jun* genes, and chromosome 8 and 15 as well. Muscles from dysferlin-deficient mice that were selected for histopathological examination shown in (C) and (D) are indicated. (B) Real-time PCR results (shown as -ΔCt values) for chromosome 8 and 15, and *Cdkn2a* revealing elevated levels of chromosome 8 and/or 15 in ∼30% and losses at the *Cdkn2a* locus in some of muscles from MD-mice but never in wild-type (WT) mice. Some of the observed values indicated relatively high fractions of cells harboring the respective alterations (indicated by filled red circles). (C) Histological examination of 3 select muscles from the genetic screen shown in (A) revealed presence of variably sized microscopic tumor masses, suggestive of well-differentiated liposarcoma, residing between muscle groups and within single muscle fascicles. (D) Histological and immunohistochemical examination of a TA-muscle sample from a 594 d-old *Dysf ^−/−^* mouse, revealing the presence of tumor cells characterized by positive staining for Cdk4 and p27.

To test this hypothesis, we assessed chromosome 8 and chromosome 15 copy numbers in DNA samples extracted from a panel of typically tumor-prone limb muscles (n = 101), which were obtained from different animals (n = 31) that were sacrificed at advanced ages (comparable to the mean age of mice with sarcomas in the respective MD models) but had not developed visible tumors until then (Figure 3A). We found elevated levels of chromosome 8 and/or 15 in ∼30% of muscles from MD-mice but never in wild-type mice (Figure 3B). Also occasional copy number aberrations of the *Cdkn2a*, *Nf1*, *Met* and *Jun* genes were detected in dystrophic muscles (∼12%). Because the extents of some of these findings were clearly compatible with the presence of malignant cell clones within the tested muscles, we next analyzed these muscles microscopically. Indeed we found variably sized microscopic tumor masses residing between muscle groups and within single muscle fascicles (Figure 3C). Immunohistochemical examination of these tumors *in situ* revealed intense staining of cell proliferation markers (p27, PCNA) as well as Cdk4 (Figure 3D), compatible with high proliferative activity.

These findings clearly showed (i) that tumor pre-stages and pre-neoplastic lesions are already present in dystrophic muscle and (ii) that the actual sarcoma incidence of MD-mice is much higher than that solely based on the occurrence of visible tumors. Because none of these DNA-abnormalities were present in non-muscle tissues (i.e. brain, liver, and lung) we concluded that these somatic aberrations are specific to dystrophic skeletal muscle.

### Recurrent patterns of somatic aneuploidy in various types of human muscular dystrophies

**Figure 4:**
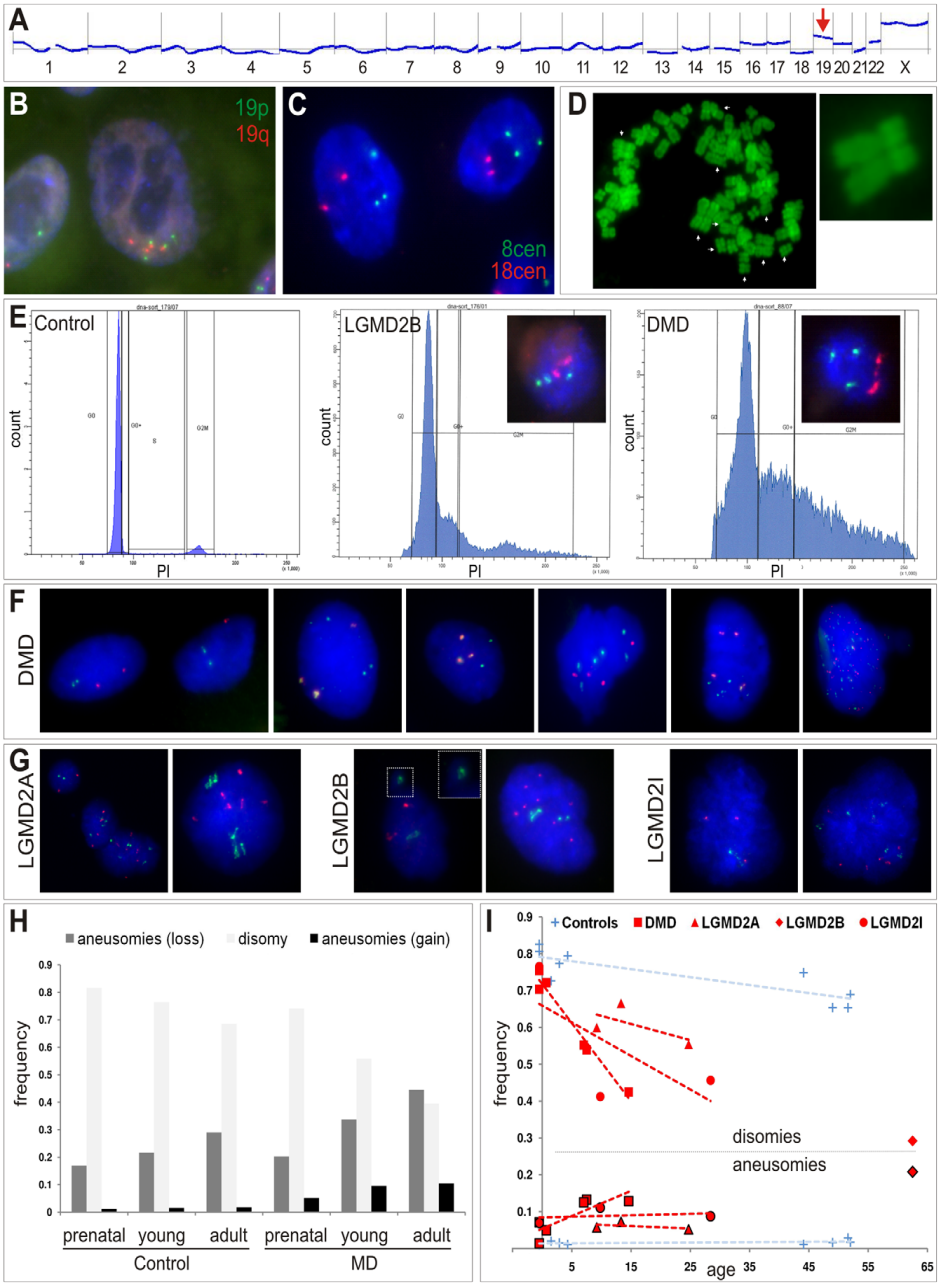
Somatic aneuploidy in human muscular dystrophies. (A) Agilent aCGH profile (50 Mbps moving average) of cultured human myoblasts from a female LGMD2B patient (“90/01”). Mean-fold changes (compared to pooled DNA from n = 6 healthy male controls) were highly suggestive for gains of several chromosomes, especially for chromosome 19 (arrow). (B,C) I-FISH on myoblasts verified the presence of nuclei with aberrant chromosome 19 (tetrasomy shown in B) and chromosome 8 counts (trisomy shown C). (D) Diplochromosome formations (arrows) in a metaphase spread prepared from DMD myoblasts. (E) DNA content analyses by FACS profiling of propidium iodide (PI)-stained myoblasts from DMD and LGMD2B patients revealed abnormally high proportions of nuclei with aberrant chromatin content indicated by highly prominent G0+ peaks compared to the control myoblast line. Insets show targeted FISH analyses of nuclei isolated through sorting G0+ peaks, which verified the presence of chromosome 8 trisomies (green: 8cen, red: 18cen). (F,G) Interphase FISH on nuclei isolated from cryofixed skeletal muscle tissue from DMD patients (F) and LGMD2A, LGMD2B and LGMD2I patients (G), probed with chromosome 2p (red) and chromosome 19q (green) probes. Close-up in one LGMD2B example depicts micronucleus formation. (H) Frequency histogram of chromosome 2p and 19q aneusomies (gains, black, and losses, dark grey) compared to the normal disomic configuration (light grey) in prenatal, young, and adult patients with MD and healthy controls. (I) Age-dependent decline of normal disomic configuration (chromosome 2p and 19q) and increase of aneusomies in skeletal muscle from MD patients (red, as indicated) in contrast to controls (blue). Outlined symbols correspond to aneusomies. Dashed lines indicate linear trends.

To address whether aneuploidy affects also human MD, we analyzed primary myoblast lines from DMD and LGMD2B patients. In myoblast DNA samples from a DMD and a LGMD2B patient as well, arrayCGH revealed profiles indicating borderline gains of several chromosomes. In particular, aberration scores indicated gains of chromosome 19 (Figure 4A). To confirm this finding, interphase fluorescent *in situ* hybridization (I-FISH) experiments were performed on cytospin preparations from early-passage myoblast cell cultures of DMD (n = 4), and LGMD2B (n = 3) patients, as well as healthy donors (n = 2). In contrast to normal cells, myoblasts from DMD and LGMD2B patients frequently harbored tetrasomies of chromosome 19 (13–27%; Figure 4B). Additional analyses for other chromosomes revealed multiple aneusomies, such as tri- and tetrasomies of chromosomes 1 (5–8%), 2 (3–6%), and 8 (4–20%; Figure 4C). In a DMD myoblast cell line, metaphase spreads displayed formation of diplochromosomes (i.e. pairs of sister chromosomes, generated by endoreduplication) (Figure 4D), which are indicative for heterogeneous chromosomal instability and aneusomies. DNA content analyses by FACS profiling of propidium iodide-stained cells revealed that myoblasts from DMD and LGMD2B patients contained abnormally high proportions of nuclei with aberrant DNA-content, indicated by prominent G0+ peaks (Figure 4E). Targeted FISH analysis of nuclei isolated through sorting of such G0+ peaks verified the presence of genomes harboring chromosome 8 aneusomies (Figure 4E, insets). Moreover, the occasional presence of micronuclei implied the continual induction of numerical or structural chromosomal damage in MD-myoblast lines.

In order to preclude that the observed chromosomal copy number aberrations had been acquired or at least amplified *in vitro,* as reported for embryonic stem cells [15] and committed progenitor cells [16], we asked whether aneusomies also represent an *in vivo* genotype and do exist in skeletal muscle tissue of MD patients. To this end, interphase nuclei from frozen muscle biopsies from human MD-patients were isolated and probed by I-FISH. We detected tri- and/or tetrasomies of chromosomes 2 and/or 19 in ∼5–12% of the nuclei isolated from DMD muscle (n = 4) (Figure 4F). In contrast, counts of chromosome 13, for which normal copy numbers were found in myoblasts, were readily comparable to control muscles (Table 2). Similarly, aberrant chromosome 2 and 19 counts were detectable in muscle biopsies from patients with LGMD2A (n = 3, *CAPN3* mutations), LGMD2I (n = 3, *FKRP* mutations), as well as LGMD2B (n = 1, *DYSF* mutations) (Figure 4G). Notably, LGMD2A muscles exhibited slightly aberrant counts also for chromosome 13 (4.6% versus 1% in controls). Generally, poly-/aneusomic nuclei further displayed features like enlargement, more irregular shape, and micronucleus formation, when compared to disomic nuclei. I-FISH signals in nuclei with aneusomic configurations frequently appeared either as highly condensed doublet signals (in particular for chromosome 19) or as bizarre structures with highly elongated conformation, indicating increased variability of differential (probably abnormal) states of chromatin condensation (Figure 4F, 4G). In order to learn if the degree of aneusomies correlates with the disease progression of muscular dystrophies, we also studied fetal muscle obtained during autopsy of aborted fetuses with prenatal diagnosis of DMD or MDC1C. Indeed, these fetal muscle tissues contained much less chromosomal copy number aberrations (chr2: ∼1% versus 0.2% in controls; chr13: 0.6% versus 0%; chr19: ∼3% versus 1%). Thus, compared to age-matched control muscles, we observed an age-dependent increase of the frequency of aneusomic nuclei in MD patients (Figure 4H, 4I).

**Table 2:** Somatic aneuploidy in human skeletal muscle from MD patients.

			disomies (%)			aneusomies (%)		
Group	Sample ID	Age (a)	chr2	chr13	chr19	chr2	chr13	chr19
***Controls***								
Control-fetal	FK	prenatal	88	91	85	0.0	0.0	1.0
Control-fetal	FP	prenatal	91	94	88	0.5	0.0	1.4
Control	M2232	2	87	81	79	0.7	1.0	1.3
Control	M1856	3	87	67	81	0.8	2.1	1.1
Control	M2066	4	91	93	84	1.1	0.0	0.7
Control -adult	M826	44	93	87	79	0.5	0.5	0.5
Control -adult	M1006	49	87	79	70	1.1	1.4	0.0
Control -adult	M983	52	87	91	70	2.3	0.0	1.1
Control -adult	M689	52	84	74	77	1.1	1.6	1.7
***DMD***								
DMD-fetal	FL	prenatal	85	93	86	2.2	0.6	4.9
DMD-fetal	FB	prenatal	83	92	81	0.0	0.5	1.4
DMD	M2006	1	82	88	84	2.7	0.0	3.3
DMD	M1994	7	71	82	70	8.3	2.4	6.8
DMD	M1895	8	76	86	66	5.9	3.6	8.8
DMD	M1959	15	58	75	54	11.2	2.0	6.1
***DYSF***								
LGMD2B-adult	M2057	62	58	n.a.	46	17.7	n.a.	7.5
***FKRP***								
MDC1C-fetal	M2166	prenatal	88	89	83	1.7	0.5	2.6
LGMD2I	M1787	10	69	81	55	7.3	1.9	6.1
LGMD2I -adult	M2190	28	62	80	62	7.1	0.7	3.3
***CAPN3***								
LGMD2A	M1883	9	77	84	66	3.9	4.1	4.6
LGMD2A	M2207	13	79	87	77	6.1	6.0	4.6
LGMD2A-adult	M2219	25	73	84	70	3.8	3.5	2.8

n.a.: not analyzed

### Widespread activation of the DNA damage response in muscular dystrophies

**Figure 5:**
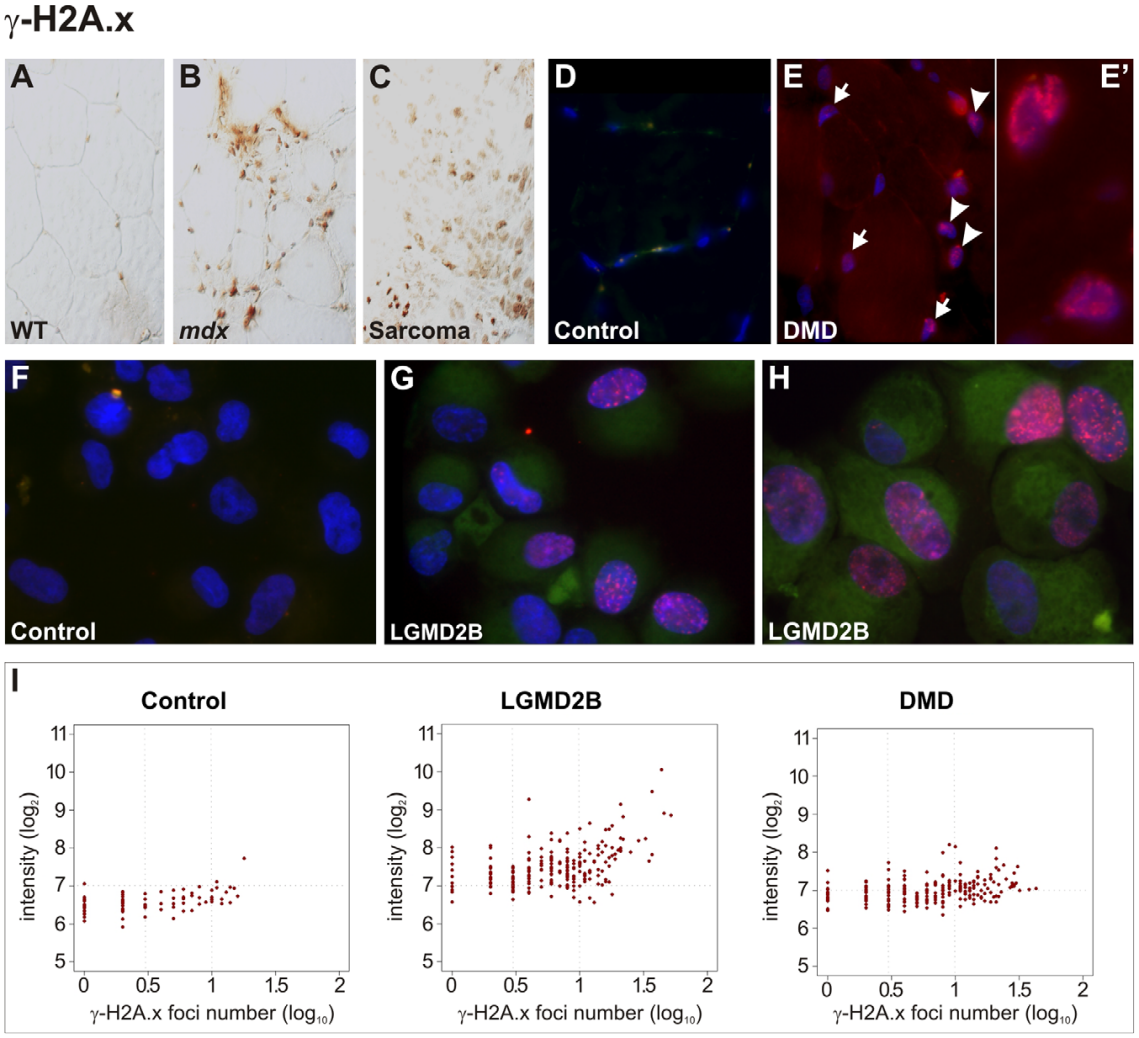
Pronounced DNA damage response in murine and human dystrophic muscle. (A–C) Intense immunoreactivity with an antibody detecting Ser139-phosphorlated histone H2A.x (γ-H2A.x) in skeletal muscle from a dystrophin-deficient (*mdx*) mouse (B) and in a sarcoma from a *mdx* mouse as well (C), in contrast to wild-type (WT) muscle (A). (D–E) In contrast to a healthy control (D), γ-H2A.x immunostainings revealed high levels of DSBs in muscle biopsies from DMD patients (E), with multiple nucleoplasmic γ-H2A.x foci formation in nuclei belonging both to muscle fibres (arrows) and non-muscle cells (arrow-heads). Close-up panel (E') shows two nuclei with intense foci formation. (F–H) Pronounced accumulation of γ-H2A.x foci in cultured myoblasts from LGMD2B patients (G, “362/03” and H, “90/01”), in contrast to myoblasts from a healthy donor (F, “363/07”). (I) Quantification of γ-H2A.x immunoreactivity revealed increased total reactivity and a prominent increase of cells with multiple (≥3) foci (log_10_  =  0.5) in myoblasts from LGMD2B (“362/03”) and DMD (“88/07”) patients compared to a control myoblast line (“363/07”). Fluorescence intensity is shown in a log_2_ scale, foci number is log_10_.

The finding of cancer-like mutations and somatic aneuploidy in dystrophic muscle prompted us to speculate that this might be caused by damage to DNA induced e.g. by oxidative or replication stress. The formation of interstitial deletions and intrachromosomal amplifications, which we found in pre-neoplastic lesions and sarcomas arising in murine MDs, belong to typical genetic aberrations that result from unrepaired DNA double-strand breaks (DSB) [17] and represent early events in the development of cancer [18]. To explore whether damage to genomic DNA precedes sarcoma development, we studied the canonical DNA damage response pathways in skeletal muscle from dystrophic mice. When analyzing muscle tissue from *Dmd ^−/−^* mice, pronounced activation of the two major DNA damage response pathways was observed, characterized by high expression of Ser1981-posphorylated ATM (p-ATM, ataxia-telangiectasia mutated kinase) and Ser428-posphorylated ATR (p-ATR, ATM and Rad3-related), and of their downstream signaling targets Chk1 and Chk2 (*not shown*). We next investigated histone H2A.x, which represents a target of the ATM pathway that signals the presence of DSBs and constitutes a key protein of the DNA damage response by accumulating at large stretches of chromatin surrounding DSBs and recruiting repair factors [19]. In contrast to normal controls, muscle from MD mice was characterized by intense immunoreactivity with an antibody specifically detecting Ser139-phosphorylated histone H2A.x (γ-H2A.x), similar to the reactivity observed in sarcomas (Figure 5A–5C).

We then examined the DNA damage response in muscle biopsies obtained from human DMD patients. In contrast to healthy control muscles, γ-H2A.x immunostainings revealed high levels of DSBs in muscle biopsies from all DMD patients (n = 4) tested, with multiple nucleoplasmic foci formation belonging to muscle fibres and moreover to non-muscle cells within the endomysial connective tissue, such as interstitial fibroblasts and endothelial cells (Figure 5D, 5E). We further found that DNA-damage response was already present in pre-pathologic muscle from very young patients (9–11 months) and a DMD fetus, which suggested that DNA-double strand breaks very likely occur prior to clinical onset of muscle weakness, wasting, and the concomitant inflammatory response. Also muscle tissue in samples from LGMD2A (*CAPN3*, n = 3), LGMD2I (*FKRP*, n = 2), MDC1C (*FKRP*), and LGMD2B patients (*DYSF*) exhibited intense γ-H2A.x immunoreactivity and multiple nucleoplasmic foci formation (*not shown*). That both muscle-fiber nuclei and non-muscle cell nuclei displayed massive γ-H2A.x accumulation prompted us to specifically assess the DSB response in myogenic precursor cells. We investigated primary muscle cell cultures generated from DMD and LGMD2B patients. In contrast to myoblasts from healthy donors, nuclei from DMD and LGMD2B myoblasts showed pronounced accumulation of γ-H2A.x foci (Figure 5F–5I). The formation of distinct nuclear immunofluorescent foci was observed in 49% of cells from DMD and 59% from LGMD2B myoblasts (compared to 24% in controls) and the number of cells with multiple (≥3) foci was also markedly increased (DMD: 32%; LGMD2B: 45%; controls: 10%).

## Discussion

### MD mice are prone to develop age-related mixed rhabdo-fibro-liposarcomas

Here we show that different types of MD mouse models develop with increasing age mixed soft-tissue sarcomas (STS), presenting as rhabdo-fibro-liposarcomas. While the spontaneous occurrence of RMS has been previously reported in *mdx* mice [20] and in addition in mice deficient of α-sarcoglycan [21] (*Sgca ^−/−^*, a model for the human LGMD2D), this is the first report of sarcomas in mice lacking dysferlin, calpain 3, or Large. Our work further shows for the first time that also mice lacking dystrophin due to other mutations than *mdx* and on different genetic backgrounds are prone to develop age-related STS. In contrast to the previous reports, we found that virtually all sarcomas from MD mice histologically present as mixed sarcomas consisting of RMS and of two additional components with fibro- and liposarcomatous differentiation. Macroscopically, sarcomas feature considerable heterogeneity regarding visual appearance and consistency of tumor mass. Similar to the high complexity and histological diversity inherent to human sarcomas, we found it extremely difficult to exactly stage individual tumors due the highly complex and heterogeneous structure and significant sectional plane divergence. Therefore, our finding of mixed sarcomas in *mdx* and other MD mice rather extends than rebuts the previous reports by Chamberlain et al. [20], who reported alveolar RMS in *mdx*, and Fernandez et al. [21], who described embryonal RMS in *mdx* and also *Sgca ^−/−^*mice. As a further difference, sarcoma incidence in our C57BL/10 *mdx* mice (39%) was clearly higher compared to the previously reported RMS incidences (∼6–9%). It remains elusive if these differences are due to different housing conditions or other unknown environmental or strain-specific factors.

It is, however, remarkable that the three main components of malignant cell-types, i.e. myo-, fibro-, and lipocytes, which we observed in our MD-mouse tumors, correspond exactly to the same cell- and tissue types that are crucially characterized by progressive proliferation in MDs. Thus, the MD-associated proliferation of fat and connective tissue might create the molecular context permitting sarcoma development arising from a multipotent mesenchymal or muscle-derived stem cell.

### MD-genes display some features similar to tumor suppressors

Several observations in our study lend support to the speculative view that MD-genes might have a role as tumor suppressors. We found that strain backgrounds with C57BL/6 proportions obviously exerted protective effects with regard to tumor latency and that tumor penetrance was lower in *Dmd ^−/−^* mice on C3H or BALB/c backgrounds compared to C57BL/10. In line with our observation, C57BL/6 is known for its resistance to *Ptch1^+/−^*-induced rhabdomyosarcomas [22]. Genetic background also clearly influenced tumor gender specificity in *Dmd* ^–^ mice (male preference in BALB/c, female in C3H) and tumor site predilection in *Dysf ^−/−^* mice (∼60% abdominal wall tumors in C57BL/10 x B6C3Fe compared to ∼20% in C57BL/10). Such strain-specific modulation of incidence, latency, location spectrum, and gender preference has been well documented for other cancer models, such as the p53-deficient mouse [23]. The significantly reduced sarcoma latency in double-mutant *Dmd ^−/−^Dysf^−/−-^* and *Dmd ^−/−^Capn3 ^−/−^* mice also resembles a common feature of tumor suppressor mouse models, as exemplified by the synergistic effect of a combined loss of p53 and Nf1, which accelerates soft-tissue sarcoma development [24]. Thus, the effects we observed for MD-gene losses represent classical credentials of tumor suppressor genes. In support of this view, dystrophin has been linked to human cancer, as its frequent inactivation was shown to be involved in the pathogenesis of malignant melanoma [25]. Notably, in melanoma cell lines dystrophin knock-down enhanced migration and invasion, whereas re-expression attenuated migration and induced a senescent phenotype, fully in line with a tumor suppressor role of dystrophin [25]. Moreover, utrophin, the highly related autosomal paralogue of dystrophin, represents a tumor suppressor candidate, owing to its frequent disruption in human malignant tumors and its capability to inhibit breast cancer cell growth [26]. Notably, aberrations of the DG have been associated with several types of human cancer [27]–[30], suggesting a potential role also in tumorigenesis. In particular, a tumor suppressor function has been suggested for laminin-binding glycans on α-dystroglycan [31], whose loss can be caused by silencing of the *LARGE* gene in several metastatic epithelial cell lines [30]. For both, dystrophin [32] and dysferlin [33] interactions with the microtubule network have been recently described, which suggests their hypothetical implication in microtubule-mediated cell functions, such as mitosis and cell migration. Future studies will be needed to clarify whether MD-genes act as tumor suppressors, which is suggested but not proven by our data.

### Recurrent non-random genetic lesions in MD sarcomas

We found that murine sarcomas from MD-mice frequently harbor non-random, recurrent genetic lesions that provide links to human mesenchymal cancers. The pivotal p53 and retinoblastoma (RB) cell cycle control pathways were frequently incapacitated by the disruption of the *Cdkn2a* locus, which encodes two different tumor suppressors, the Cdk4 kinase inhibitor p16^INK4a^ and the Mdm2-p53 regulator p19^ARF^, both of which play an important role in the development and progression of many human cancer types. Deletions at *Trp53* and *Nf1* loci established a genetic link to human soft-tissue sarcomas, which are characterized by frequent p53 mutations [34]–[36], as well as to syndromes associated with increased RMS incidence due to germ-line disruption of these tumor suppressor genes (Li-Fraumeni, *TP53*; Neurofibromatosis type I, *NF1*) [37]. More recently, human myxofibrosarcoma and pleomorphic liposarcomas were shown to frequently harbor *NF1* mutations [14]. Thus, the disruption of *Nf1* in sarcomas from MD-mice parallels specific - non myogenic - subtypes of human soft-tissue sarcomas and suggests a more general role for *Nf1-*lesions in the genesis of mesenchymal cancers. A high fraction of sarcomas from MD-mice harbored amplifications of the *Met* or *Jun* oncogenes. The *Met* oncogene amplification constitutes a critical path to aberrant activation of the Hgf/c-Met axis, which is known to promote tumorigenesis and to be involved in the progression and spread of multiple human cancers. Amplification of the *JUN* oncogene has been reported in human liposarcomas [38]–[39], in sound accordance with herein discovered frequent *Jun* amplification in MD mixed sarcomas.

Our finding of recurrent chromosome 8 and/or 15 gains in MD sarcomas provides a link to other murine cancers. Chromosomes 8 and/or 15 are frequently duplicated in T cell tumors [40]–[41] or transgenic mouse models of acute promyelocytic leukemia [42], and probably contribute to elevated expression of the *Junb* and/or *Myc* oncogenes, as suggested for *Myc* in T cell lymphomas [41]. Notably, several human malignancies, amongst them myxoid/round cell liposarcoma [14], are known to harbor recurrent gains of chromosome 8. Most importantly, the human chromosome 8 harbors multiple regions that are syntenic to both murine chromosomes 8 and 15, which we found to be regularly gained in the MD-sarcomas.

### Dystrophic skeletal muscle from mice and humans harbors cancer signature, somatic aneuploidy, and DNA damage

We discovered that the most frequent and most prominent genetic alterations that characterize full-blown skeletal muscle-derived sarcomas are already present in dystrophic skeletal muscle of clinically tumor-free mice. We also demonstrated DNA damage and showed that skeletal muscle of MD-mice harbors microscopic tumor infiltrates prior to the development of macroscopically visible tumors. In particular, our findings suggested that somatic aneuploidy, indicated by recurrent gains of chromosomes 8 and 15, contributes to sarcoma susceptibility in murine MD. Thus, the frequent occurrence of chromosome 8/15 gains together with specific losses at the *Cdkn2a* locus might represent early events occurring in cancer pre-stages and promoting malignant transformation [43]. Importantly, these findings also suggested that the actual sarcoma incidence of MD-mice is much higher than that solely based on the occurrence of visible tumors. In the light of our results, sarcoma formation might be regarded as the disease end-stage of a MD in mice.

The finding of cancer-like genomic aberrations and DNA damage in the skeletal muscle from MD-mice inspired us to search for such aberrations in skeletal muscle of human MD patients. We focused on DMD and LGMDs caused by *DYSF*, *CAPN3*, or *FKRP* mutations, representing the most frequent MDs, and found that all of them are associated with somatic aneuploidy and widespread DNA damage in skeletal muscle tissue *in vivo*. Also *in vitro*, cultured myogenic stem cells from DMD and LGMD2B patients exhibited DNA damage and aneuploidy. In our study, somatic aneuploidy appeared to be a feature concurring with the outbreak of pathology in dystrophic muscle and to increase with age in human MD patients. In contrast, high levels of DSBs were already evident in fetal muscle from DMD and MDC1C individuals and in muscle biopsies from DMD infants (<1 year), which suggested that DNA damage precedes the clinical manifestation and therefore cannot be solely related to replication stress. While somatic aneuploidy has been reported in multiple human pathologies, such as Alzheimer's disease, this is the first report on gross somatic aneuploidy in MDs. Genomic instability has been reported in laminopathy-based premature ageing [44], a condition caused by mutations in lamin A/C, notably another MD-related molecule. DNA damage was shown recently in Friedreich's ataxia, a neurodegenerative disorder [45].

Depending on the context, aneuploidy not only can promote tumorigenesis [18], [46]–[47], but also can impair proliferation, cause premature replicative senescence [48], or can even suppress tumorigenesis [49]. Under the assumption that aneuploidy affects cells destined for muscle regeneration and/or function, aneuploidy could therefore represent an important pathological feature causing a propensity for malignant transformation in murine MDs and contributing to tissue malfunction and diminished regenerative capacity in human MDs. Unrepaired DNA damage activates cellular senescence [50] and could therefore be also associated with the known generalized diminished replicative capacity of DMD myoblasts [51], contributing to the progressive exhaustion of the muscle's regenerative potential [52]. In the murine condition, senescence could also underlie sarcoma susceptibility as secreted senescence associated factors can contribute to a pro-tumourigenic inflammatory environment [50], thereby promoting the occurrence of age-related cancer [53]. In this context, it will be interesting to study sarcoma formation in *mdx* mice lacking the RNA component of telomerase (mdx/mTR) that have very recently been shown to have shortened telomeres in muscle cells and a severe progressive muscular dystrophy [52].

For the time being, we have no answer for why murine MDs frequently end up in sarcoma formation while in human MD patients increased muscle-tumor susceptibility has not been reported. But it is interesting to note that it is also not fully understood why loss of dystrophin causes a fatal MD in humans while only a mild myopathy in mice. Also, *Dysf ^−/−^* and *Capn3 ^−/−^* deficient mice are largely spared the severe symptoms of the patients with LGMD due to defects in these two genes. We speculate, however, that essential differences in tumor biology between men and mice could account for this difference: while humans are prone to epithelial carcinomas, mice commonly develop mesenchymal sarcomas, which might be due to profound differences in telomere biology between the two species [54]. Also, fewer genetic events are required to induce malignant transformation in mice compared to humans [43], [55].

### Concluding remarks

Collectively, our findings that genetically distinct MDs in mice and humans share a common molecular pathology characterized by DNA damage and genomic instability similar to pre-cancerous lesions suggests the existence of a novel, unifying pathomechanism that might contribute to disease progression through erosion of the replicative capacity of muscle stem cells and could therefore help to explain the common fatal progression of degeneration and wasting in MD. This is a novel aspect, which contributes to our understanding of MD, and moves an orphan disease close to the common disease cancer, thereby hopefully opening novel therapeutic avenues.

## Materials and Methods

### Patients and muscle tissue biopsies

Samples for this study were collected from diagnostic skeletal muscle biopsies, which had been conducted in patients assigned for evaluation of musculoskeletal disorders at our department. Patients or their legal guardians gave informed consent for scientific purpose use of left-over tissue samples. DMD patients included in this study had a confirmed molecular diagnosis of DMD, ascertained by lack of dystrophin staining in immunohistochemistry (IH) and Western Blot (WB), and in most cases a genetic diagnosis. Muscle biopsy samples used in this study were from a total of n = 6 different DMD patients: M2006 (age at muscle biopsy: 9 m; *DMD* gene mutation: c.3053_3087del), M2008 (11 m; c.8669-1G>T), M1633 (6 a; c.858T>G p.Tyr286X), M1994 (7a; unknown *DMD* mutation), M1895 (8 a; dup_ex3-7), M1959 (15 a; del_ex17), and two samples from aborted fetuses with DMD. LGM2DA patients (n = 3) had a genetic diagnosis and WB exhibited absence of calpain-3 specific bands in muscle tissue: M1883 (9 a; c.550delA p.Thr184ArgfsX36), M2207 (13 a; c.550delA), M2219 (25 a; c.1342C>T p.Arg448Cys). LGMD2I patients (n = 3) had a confirmed diagnosis by *FKRP* gene sequencing: M1787 (10 a; c.854A>C p.Glu285Ala), M2190 (28 a; c.826C>A, p.Leu276Ile), M2166 (prenatal; c. [962C>A]+[1086C>G] p.Ala321Glu + p.Asp362Glu). LGMD2B in one patient was confirmed by reduced dysferlin reactivity in IH and WB, and *DYSF* gene sequencing: M2057 (62 a, c.509C>A p.Ala170Glu). All tissue samples were snap-frozen in dry ice-cooled 2-methylbutane within 1 h after biopsy and stored at -80°C until use.

### Myoblasts

Primary myoblast cultures were obtained from the Muscle Tissue Culture Collection, Friedrich-Baur-Institute, Department of Neurology, Ludwig-Maximilians-University Munich (Germany). DMD: “Essen 88/07” (14 a, del45_50); “72/05” (7 a, dup_ex8-29); “Essen 8/02” (4 a, del_ex51-55); “166/00” (6 a, 2bp-deletion in exon 6); LGMD2B: “90/01” (36 a, female, c. [638C>T]+ [5249delG]); “176/01” (32 a, male, c. [2367C>A]+ [5979dupA]); “362/03” (male, 33 a, c. [exon 5 p.Pro134Leu]+ [5022delT]); controls: “363/07” (21a, male); “179/07” (21a, female). Cells were maintained in Ham's F-12 medium supplemented with 15% fetal bovine serum, GlutaMax (L-glutamine 200 mM), glucose (6.6 mM), fetuin (0.47 mg/mL), bovine serum albumin (0.47 mg/mL), dexamethasone (0.38 µg/mL), insulin (0.2 µg/mL), epidermal growth factor (10 ng/mL), Pen-Strep (penicillin G 5000 units/mL, streptomycin 5 mg/mL), and fungizone (amphotericine B 0.5 µg/mL) at 37°C in a humidified atmosphere of 5% CO_2_: 95% air. For experimental purposes, cells were harvested after 3 or 4 passages. DNA was isolated using the QIAamp DNA Mini Kit (Qiagen, Hilden, Germany) according to the manufacturer's recommendations. RNA was isolated using TRI Reagent (Sigma-Aldrich, St. Louis, MO). Cells were stained with BD Cycletest Plus, DNA Reagent Kit for DNA content analysis by flow cytometry (BD Biosciences, San Jose, CA).

### Mice

Mice stocks were maintained at the Division for Laboratory Animal Science and Genetics (Medical University Vienna, Himberg, Lower Austria) under institutionally approved protocols for the humane treatment of animals. Mice were cared for in our facilities under conventional housing conditions and received food and tap water *ad libitum*. Mice presenting with weakness received intensive care, were fed with food pellets soaked in tap water, and were examined daily. Aged mice were checked daily for the development of tumors. In general, tumors were characterized by rapid growth, necessitating the killing of affected mice within few days after visual identification of sarcomas. The *Dmd^mdx^* C57BL/10 (*mdx*), *Dmd^mdx-3cv^* C57BL/6 (*mdx-3cv*), SJL *Dysf^im^* (*SJL-Dysf*), and B6C3Fe *Large^myd^* (*myd*) mice were originally obtained from The Jackson Laboratory (Bar Harbor, ME). To study dystrophin deficiency on other strains, we inbred the *Dmd^mdx^* mutation to C3H and BALB/c (>20 consecutive backcross generations; residual heterozygosity <0.01). Some *mdx* mice were maintained on a mixed C57BL/6 x BALB/c background. Further, we inbred the *SJL-Dysf* mutation onto the C57BL/10 background, where a prolonged life span compared to *SJL* was observed, which enabled us to study late-onset stages of dysferlin-deficiency. *Capn3* knockout mice (*Capn3^tm1Jsb^*) [11] on the 129/Sv x C57BL/6 background were obtained from Isabelle Richard and crossed to C57BL/6 mice. To generate *Dmd ^−/−^ Dysf ^−/−^* and *Dmd ^−/−^ Capn3 ^−/−^* double-mutants, *mdx* mice were crossed to *SJL-Dysf* (C57BL/10) and *Capn3* knockout mice. Some *mdx* C57BL/10, *mdx-3cv* C57BL/6, as well as *SJL-dysf* C57BL/10 mice were crossed to B6C3Fe *myd* mice. Of these animals, only *Large+/−* and *Large+/+* mice were used for analysis of tumorigenesis, which were indistinguishable from pure *mdx*, *mdx-3cv*, and *SJL-dysf* mice, respectively. In all cases, *Large+/−* heterozygosity had no influence on tumorigenesis and conferred no overt additional phenotype with regard to muscle pathology. After sacrifice by cervical dislocation, mice were dissected, and muscles, other tissues, and (where applicable) tumors were excised and snap-frozen in dry ice-cooled 2-methylbutane. All samples were stored at -80°C.

### Tumor cell cultures

Using sterile techniques, parts of excised tumors were washed in PBS, cut into small pieces, and cultured in primary medium, containing DMEM (Dulbecco's Modified Eagle's Medium, 4.5 g/L glucose; PAA Laboratories, Pasching, Austria), 20% fetal bovine serum (FBS “GOLD” Origin: USA; PAA Laboratories), 200 U/l PenStrep (Penicillin, Streptomycin; Lonza, Cologne, Germany) and 2.5 µg/ml Fungizone (Gibco, Invitrogen Ltd, Paisley, UK). After sporadic adhesion of tumor cells, remaining tissue parts were removed, and the primary medium was replaced by growth medium (DMEM, 20% FBS, 50 U/l PenStrep). To activate differentiation, FBS was replaced by 2% horse serum (PAA Laboratories). DNA/RNA extraction was performed as described above for cultured myoblasts.

### DNA and RNA isolation from mouse tissues

DNA was isolated from serial 5 µm-cryosections prepared from dissected skeletal muscle (∼5 mg) or tumor (∼10 mg) specimens. Reference sections from the sampling procedure were HE-stained for histomorphological examination. Sections were stored at −80°C and then subjected to tissue lysis and nucleic acid purification according to the QIAamp DNA Mini Kit protocol (Qiagen). Mouse tail DNA was isolated using the same protocol starting from lysates prepared by directly lysing 2–3 mm tail tips. DNA concentrations were measured using the NanoDrop spectrophotometer (Peqlab, Erlangen, Germany), DNA samples were diluted (10 ng/µl) and stored at −20°C until use. RNA was extracted from serial 10 µm-cryosections by lysis in 1 TRI Reagent (Sigma-Aldrich), chloroform extraction, and precipitation with isopropanol. RNA samples were measured by spectrophotometry (NanoDrop) and quality controlled using BioAnalyzer LabChips (Agilent Technologies, Santa Clara, CA).

### Histology and immunohistochemistry

Cryosections were stained with haematoxylin and eosin (HE). Sudan Black B was used for lipid staining. For immunohistochemistry, 10 µm cryosections were fixed using 3.7% paraformaldehyde (5 min), treated with 0.1% Triton-X100 (5 min), rinsed in PBS, and subsequently incubated with primary antibodies. For immunocytochemistry, cytospins were prepared from cell suspensions and subjected to methanol/acetic acid (3:1) fixation before antibody incubation. Primary antibodies used in this study were as follows: Myogenin (Santa Cruz Biotechnology, CA; sc-576), Myf-5 (sc-302), desmin (Millipore, Billerica, MA, MAB3430), Cdk4 (sc-260), PCNA (sc-7907), p27 (sc-776), p-Ser1981-ATM (Cell Signaling Technology, Danvers, MA; #4526), phopsho-Ser428-ATR (#2853), p-Ser296-Chk1 (#2349), p-Thr68-Chk2 (#2661), p-Ser139-Histone H2A.X (#9718). Secondary antibodies were conjugated to Alexa-Fluor 488, Alexa-Fluor 594 (Molecular Probes Invitrogen, Carlsbad, CA), Cy3 (Dianova, Hamburg, Germany), or to horseradish peroxidase. Where indicated, immunostained sections were counterstained with 4′,6-diamidino-2-phenylindole (DAPI) and then analyzed by confocal microscopy using either Olympus Fluoview or Zeiss Axioplan2 microscopes. Fully automated software-assisted quantification of DNA damage (γ-H2A.x foci) in myoblasts was performed using the software Metafer (MetaSystems, Altlussheim, Germany). Graphical representations (plots of fluorescence intensity versus foci numbers) were generated in R.

### Array comparative genomic hybridization (aCGH)

Matched pairs of sarcoma and tail-tip DNA (as reference) samples from the same mice were analyzed using the Agilent mouse genome CGH 44K (design ID 015028) and 244K (014695) oligonucleotide microarrays (Agilent Technologies). Human myoblast DNA samples were analyzed on Agilent human genome CGH 44K arrays (014950), using as reference human genomic DNA from multiple anonymous male donors that was purchased from Promega (G147A; Madison, WI). Labeling and hybridization procedures were performed according to the instructions provided by Agilent. In brief, 200 ng of test and reference DNA were digested with *Alu*I and *Rsa*I (both Promega) and then subjected to differential labeling by random priming with incorporation of either Cyanine 3- or Cyanine 5-dUTP (PerkinElmer, Waltham, MA) using the BioPrime Array CGH Genomic Labeling System (Invitrogen, Carlsbad, CA). After purification with Microcon YM-30 centrifugal filter units (Millipore), the labeled products were combined, mixed with blocking agent, Hi-RPM hybridization buffer (both included in the Oligo aCGH/ChIP-on-Chip Hybridization Kit, Agilent), human Cot-1 DNA (Roche Diagnostics, Mannheim, Germany) or mouse Cot-1 DNA (Invitrogen), and hybridized onto respective microarray slides. Hybridization was carried out for 48 h at 65°C in a hybridization oven. Slides were washed according to the protocol by Agilent, scanned using the Agilent Technologies Scanner G2505B and analyzed using the Feature Extraction and Genomic Workbench 5.01 (formerly DNA Analytics 4.0) software.

### Quantitative PCR (qPCR)

To screen for *Met* (chromosome [chr] 6) and/or *Jun* (chr 4) as well as *Cdk4* and/or *Mdm2* (chr 10) oncogene amplification, tumor DNA samples (25 ng) were subjected to a quantitative endpoint PCR, consisting of 0.4 µM each primer, 0.2 mM dNTPs, 1.5 mM MgCl_2_, (NH_4_)_2_SO_4_-containing amplification buffer, and 0.5 units *Taq* DNA polymerase (reagents from Fermentas, St. Leon-Rot, Germany). Competitive co-amplification of internal control targets (with similar amplicon size) allowed the unambiguous determination of ≥4-fold amplification levels. Primer sequences (5′->3′) were as follows: Met_f AAC TGT TCT TGG AAA AGT GAT CGT; Met_r TTT GAA ACC ATC TCT GTA GTT GGA; S100a8_f CGT TTG AAA GGA AAT CTT TCG TGA; S100A8_r TAT CCA GGG ACC CAG CCC TA; Jun_f AAA GCA GAC ACT TTG GTT GAA AG; Jun_r CGC TAT TAT AAA TAT GCA CAA GCA A; Mdm2_f CAT CGC TGA GTG AGA GCA GA; Mdm2_r AAG ATG AAG GTT TCT CTT CTG GTG; Cdk4_f AGT TTC TAA GCG GCC TGG AT; Cdk4_r TCT CTG CAA AGA TAC AGC CAA C; Lig3_f AGG AGA GAA GCT GGC TGT GA; Lig3_r AGC TTT CCT TCC TCT TTG CC. After cycling (3 min 95°C, 30× [40 sec 95°C, 40 sec 60°C, 1 min 72°C], 3 min 72°C), 5 µl aliquots of reaction products were analyzed on ethidium bromide-stained 1.5% agarose gels and quantified from captured images using Image J. Relative *Met*, *Jun*, *Cdk4*, and *Mdm2* copy number levels were calculated by normalization to the internal standard (*S100a8* on chr3 and *Lig3* on chr11, respectively). Tumor samples with copy numbers indicating oncogene amplification were also subjected to verification by real-time PCR (see below). Deletions at the *Cdkn2a* (chr 4) and *Nf1* (chr 11) loci were measured using a quantitative real-time PCR (qRT-PCR) SybrGreen assay (ΔCt method), involving separate amplification of target genes and an internal reference (*Lig3*). Primers were designed for *Cdkn2a* exon 2, which encodes parts of both p16^INK4a^ and p19^ARF^. CGH 244K data from one *Dysf^ -/-^* tumor revealed a compound loss at the *Nf1* locus consisting of a large ∼0.4 Mbp deletion encompassing the whole gene and a smaller ∼42 kb deletion spanning exons 9–28. To screen for *Nf1* deletions in other tumors, two different exons (23 and 56) were chosen as qRT-PCR targets. Primer sequences were as follows: Cdkn2a_f: GTA GCA GCT CTT CTG CTC AAC TAC; Cdkn2a_r AAT ATC GCA CGA TGT CTT GAT GT; Nf1_I22_f TGA TGA AGT AGT TTG CCA TTG TTT; Nf1_E23_r TTG CCA TCA TGA CTT CAA CTA ACT; Nf1_I55_f CTC TCG CTC TTC ATT TCA TCT TCT; Nf1_E56_r GCC ATA AGC CAT TAA AAC CAA AAC. *Met* and *Jun* targets were the same as above. 15 ng of DNA template were amplified in the presence of 0.5 µM primers and components of the SensiMixPlus SYBR universal mix (Quantace, London, UK) using the Stratagene Mx3005P cycler (Agilent). Cycling conditions: 10 min 95°C, 40× [40 sec 95°C, 40 sec 60°C, 1 min 72°C], followed by a dissociation segment for melting curve analysis. Chromosome 8 and 15 gains were also assessed by qRT-PCR choosing *Junb* (chr 8) and *Myc* (chr 15) as targets, respectively. Gene dosage was normalized to an arbitrary gene on chr 12 (*Prima1*), whose copy number appeared widely stable in the CGH screen. Primer sequences were as follows: Junb_f GCA GCT ACT TTT CGG GTC AG; Junb_r GTG GTT CAT CTT GTG CAG GTC; Myc_f CCA CCT CCA GCC TGT ACC T; Myc_r GTG TCT CCT CAT GCA GCA CTA; Prima1_f GTT TCC ATA TCT GCA GGT GAC A; Prima1_r CTC TCG TTC ATC AGC TGT TCC T. Reactions were carried out as above but with 30 sec extension steps. Fluorescence data were analyzed using the MxPro 4.1 software (Stratagene). After verification of primer performance, relative quantification was obtained using the threshold cycle method; ΔCt values were calibrated to wild-type (C57BL/10) tail-tip DNA. Plots of ΔΔCt (sarcomas) values were done in R and graphical representations of ΔCt values from skeletal muscle DNA samples were made in Microsoft Excel 2007.

### Reverse transcriptase PCR (RT-PCR)

To study whether mixed sarcomas from MD-mice express select human sarcoma-related genes, we subjected RNA isolated from primary tumor samples as well as from tumor cell cultures to quantitative RT-PCR. Total RNA (1 µg) was reverse-transcribed by standard oligo-dT primed cDNA synthesis using M-MuLV Reverse Transcriptase in a reaction buffer containing 50 mM Tris-HCl (pH 8.3 at 25°C), 50 mM KCl, 4 mM MgCl_2_, 10 mM DTT, and 1 mM dNTPs (Fermentas). An aliquot corresponding to 10 ng of the initial RNA sample was subjected to a quantitative endpoint PCR, consisting of 0.4 µM each primer, 0.2 mM dNTPs, 2 mM MgCl_2_, (NH_4_)_2_SO_4_-containing amplification buffer, and 0.25 units DreamTaq Green DNA Polymerase (reagents from Fermentas) in a 25 µl reaction volume. Primer sequences for the human rhabdomyosarcoma-marker genes (*Myog*, *Myl4*, *Igf2, Prox1*), a fibrosarcoma gene (*Vcan*), and liposarcoma-related genes (*Pparg*, *Myo1e*, *Hoxa5*, *Plau*) are available from the authors on request. After cycling (3 min 95°C, 35× [20 sec 95°C, 20 sec 60°C, 40 sec 72°C], 3 min 72°C), 10 µl aliquots of reaction products were analyzed on ethidium bromide-stained 1.5% agarose gels and quantified from captured images using Image J. Relative RNA abundance was calculated by normalization to the *Gapdh* transcript levels and compared to skeletal muscle samples isolated from wild-type and *mdx* mice. RNA abundance in tumor cell lines was compared to murine C2C12 myoblast cells. Visualization of gene expression was accomplished by heatmaps made in R using the heatmap.2 function.

### Interphase fluorescence *in situ* hybridization (I-FISH)

For I-FISH experiments on myoblasts, cells were fixed using 4% formaldehyde. FISH analysis on interphase nuclei extracted from cryofixed tissues was performed according to a previously published protocol with modifications [56]. In brief, thirty 20 µm-cryosections were fixed in PBS-buffered 4% paraformaldehyde (2–3 h at ambient temperature), rinsed twice with 0.9% NaCl and stored at 4°C overnight until further use. Fixed tissue sections were then transferred into a 90 µm nylon mesh and subjected to proteinase K digestion (0.05%; 10–15 min 37°C). After harvesting by cytospinning through the mesh, nuclei were air-dried, fixed with paraformaldehyde solution (4% in PBS), washed with 1× PBS (2×3 min), pre-treated with sodium thiocyanate (1 M, 80°C 1 min), and subjected to digestion with proteinase K (1 min at 37°C). After fixation, slides were air-dried, followed by heating to 78°C (8 min) for denaturing. Slides were then incubated with digoxigenin or biotin labeled chromosome probes (2p, 18cen, 19q from Dr. M. Rocchi, Molecular Cytogenetic Resource Centre, Bari, Italy; chr1 from Dr. Howard J. Cooke [57]; 8cen purchased from Kreatech Diagnostics, Amsterdam, The Netherlands; chr13 *FKHR* and 19p/19q from Vysis, Abbott Laboratories, IL) for hybridization overnight at 37°C. Slides were washed in 2× SSC 50% formamide, and 2× SSC at 42°C, and incubated with Cy3-labelled anti-biotin (Dianova, Hamburg, Germany) or FITC-labeled anti-digoxigenin antibodies in 2% BSA for 30 min at 37°C in a humid chamber. After washing in 4× SSC 0.1% Tween-20 (2×7 min at 42°C), slides were incubated with secondary antibodies labeled with Cy3 or FITC (Dianova) in 2% BSA for 30 min at 37°C, washed again as above, ethanol-dried, and mounted using Vectashield with DAPI (Vector Laboratories, Burlingame, CA). Slides were analyzed using an Axioplan2 (Zeiss) microscope and I-FISH signals were captured using the ISIS software and quantification of the I-FISH spots was achieved with the Metafer software (both from, MetaSystems, Altlussheim, Germany). For each sample 300 nuclei were automatically detected by the software and subsequently visually inspected by two independent investigators. Data presented were calculated from an average of 200 nuclei eligible for analysis.

## Supporting Information

Figure S1 Expression of myogenic and human sarcoma biomarker genes in murine MD mixed sarcomas. To study whether mixed sarcomas from MD-mice express select human sarcoma-related genes (rhabdomyosarcoma-marker genes: *Myog*, *Myl4*, *Igf2*, *Prox1*, a fibrosarcoma gene: *Vcan*, and liposarcoma-related genes: *Pparg*, *Myo1e*, *Hoxa5*, *Plau*), we subjected RNA isolated from primary tumor samples as well as from tumor cell cultures to quantitative RT-PCR. The figure shows a heatmap representation of expression levels corresponding to human sarcoma-related genes, revealing high abundance of not only rhabdomyosarcoma (Rhabdo) marker genes but also of genes related to human fibrosarcoma (Fibro) and liposarcoma (Lipo) in both, primary sarcomas (A) and *in vitro* tumor cell cultures (B).
(PDF)
