# Supplementary figures and images for: DNA Damage, Somatic Aneuploidy, and Malignant Sarcoma Susceptibility in Muscular Dystrophies
Source: PLoS Genet. 2011 Apr 14;7(4):e1002042. doi: 10.1371/journal.pgen.1002042 (PMC3077392; doi:10.1371/journal.pgen.1002042)

**A**

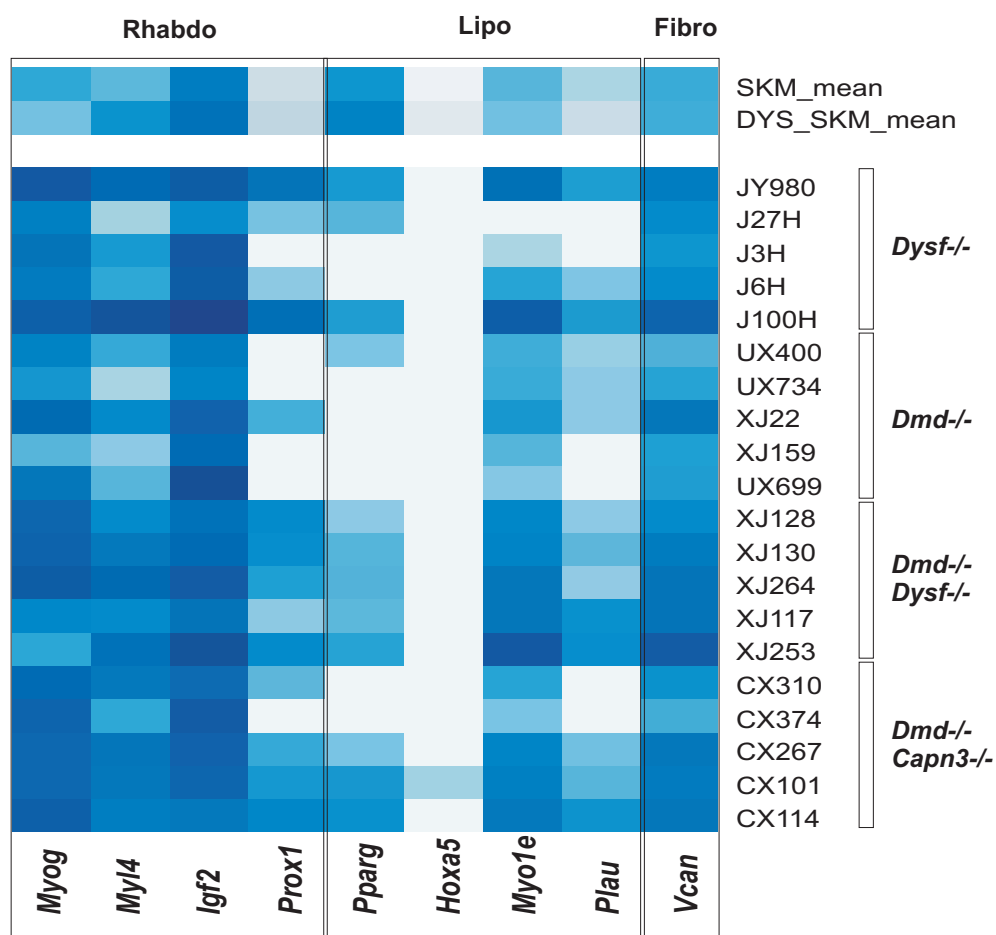

**B**

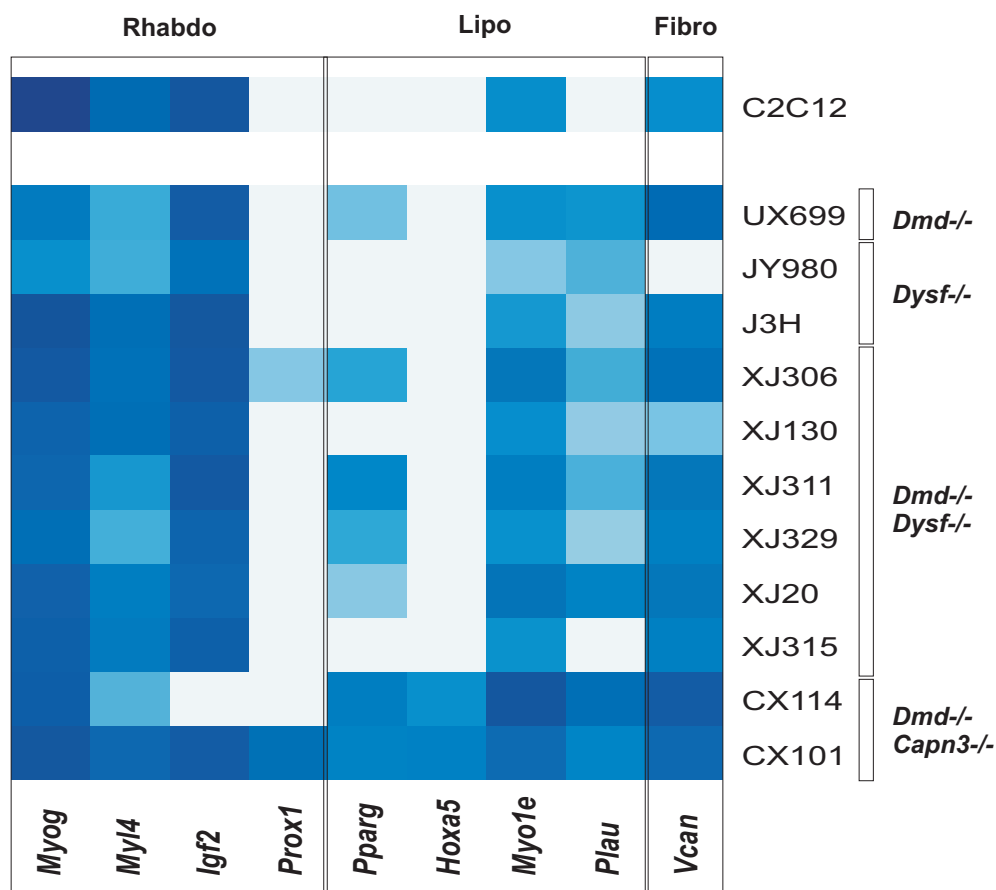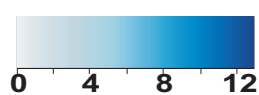

Supplement: Figure S1 — Expression of myogenic and human sarcoma biomarker genes in murine MD mixed sarcomas. To study whether mixed sarcomas from MD-mice express select human sarcoma-related genes (rhabdomyosarcoma-marker genes: Myog, Myl4, Igf2, Prox1, a fibrosarcoma gene: Vcan, and liposarcoma-related genes: Pparg, Myo1e, Hoxa5, Plau), we subjected RNA isolated from primary tumor samples as well as from tumor cell cultures to quantitative RT-PCR. The figure shows a heatmap representation of expression levels corresponding to human sarcoma-related genes, revealing high abundance of not only rhabdomyosarcoma (Rhabdo) marker genes but also of genes related to human fibrosarcoma (Fibro) and liposarcoma (Lipo) in both, primary sarcomas (A) and in vitro tumor cell cultures (B). (PDF) [file pgen.1002042.s001.pdf]
